# Colorectal cancer stage at diagnosis in migrants versus non-migrants (KoMigra): study protocol of a cross-sectional study in Germany

**DOI:** 10.1186/1471-2407-14-123

**Published:** 2014-02-24

**Authors:** Anne Dahlhaus, Corina Guethlin, Arthur Schall, Maja Taubenroth, Reyn van Ewijk, Hajo Zeeb, Zeycan Albay, Sylvia Schulz-Rothe, Martin Beyer, Ferdinand M Gerlach, Maria Blettner, Andrea Siebenhofer

**Affiliations:** 1 Institute of General Practice, Goethe-University Frankfurt, Frankfurt am Main, Germany; 2 German Cancer Research Center (DKFZ), Heidelberg, Germany; 3 German Cancer Consortium (DKTK), Heidelberg, Germany; 4 Institute of Medical Biostatistics, Epidemiology and Informatics (IMBEI), Johannes Gutenberg University Mainz, Mainz, Germany; 5 Leibniz Institute for Prevention Research and Epidemiology - BIPS GmbH, Bremen, Germany

**Keywords:** Colorectal cancer, Cross-sectional, Observational study, Ethnicity, Hard-to-reach population, Health care access, Migrants

## Abstract

**Background:**

In Germany, about 20% of the total population have a migration background. Differences exist between migrants and non-migrants in terms of health care access and utilisation. Colorectal cancer is the second most common malignant tumour in Germany, and incidence, staging and survival chances depend, amongst other things, on ethnicity and lifestyle. The current study investigates whether stage at diagnosis differs between migrants and non-migrants with colorectal cancer in an area of high migration and attempts to identify factors that can explain any differences.

**Methods/Design:**

Data on tumour and migration status will be collected for 1,200 consecutive patients that have received a new, histologically verified diagnosis of colorectal cancer in a high migration area in Germany in the previous three months. The recruitment process is expected to take 16 months and will include gastroenterological private practices and certified centres for intestinal diseases. Descriptive and analytical analysis will be performed: the distribution of variables for migrants versus non-migrants and participants versus non-participants will be analysed using appropriate *χ*2-, t-, F- or Wilcoxon tests. Multivariable, logistic regression models will be performed, with the dependent variable being the dichotomized stage of the tumour (UICC stage I versus more advanced than UICC stage I). Odds ratios and associated 95%-confidence intervals will be calculated. Furthermore, ordered logistic regression models will be estimated, with the exact stage of the tumour at diagnosis as the dependent variable. Predictors used in the ordered logistic regression will be patient characteristics that are specific to migrants as well as patient characteristics that are not. Interaction models will be estimated in order to investigate whether the effects of patient characteristics on stage of tumour at the time of the initial diagnosis is different in migrants, compared to non-migrants.

**Discussion:**

An association of migration status or other socioeconomic variables with stage at diagnosis of colorectal cancer would be an important finding with respect to equal health care access among migrants. It would point to access barriers or different symptom appraisal and, in the long term, could contribute to the development of new health care concepts for migrants.

**Trial registration:**

German Clinical Trials Register DRKS00005056.

## Background

Around 16 million people with a so-called migration background currently live in the Federal Republic of Germany [1], corresponding to almost 20% of the total population. This includes foreign nationals, but also naturalised migrants and those born in Germany with at least one parent who migrated (all referred to as migrants in the text) [2]. Migrants have entered Germany since World War II, including late-migrants from the collapsing Union of Soviet Socialist Republics, immigrating labourers (mainly from Turkey, Greece, Italy and Spain), and also fugitives or asylum-seekers from very different countries in the world.

In addition to integration through language, work and social life, access to appropriate health care is essential for migrants. With the National Integration Plan, the Federal Government has enshrined such integration in its politics [3]. Compared to the rest of the population, migrants are often worse off from a social perspective [4]. Educational background, as well as a person’s living and working situation all influence the risk of illness, and in turn health problems have a detrimental effect on educational and job opportunities [4]. Differences also exist between migrants and non-migrants in terms of health care. Migrants take advantage of prevention and early detection programmes less often than non-migrants and consult doctors less frequently [5]. This may be because some migrant populations are less familiar with the concept of routine screening to detect health problems before the onset of symptoms [6,7].

Studies from other countries report that incidence, stage and survival chances depend, amongst other things, on ethnicity [7-10]. A recent epidemiological review reports that age-standardised mortality and the incidence of gastrointestinal tumours are lower among migrants [11]. This may be partially attributable to selection effects: lower average age, lower exposure to colorectal cancer (CRC) risk factors (‘Mediterranean diet’, less alcohol), the ‘healthy migrant effect’ etc. Nevertheless, tumours are among the leading causes of death among migrants [11].

There are more than 65,000 new cases of and 25,000 deaths from CRC in Germany per year, making it the second most common malignant tumour [12]. The cumulative lifetime risk of developing CRC is 6.1% for women and 7.5% for men [12]. Median age at diagnosis is 71 years for men and 75 years for women [12], and many first generation migrants in Germany are now entering this age range.

As the CRC survival rate depends on the stage of the tumour at diagnosis [13,14], and late-stage CRC diagnoses result in higher health costs [15,16], early detection is important. It is therefore necessary to examine whether migrants are generally diagnosed at a similar stage of CRC and, apart from migration status, what further predictors of late-stage CRC should be taken into account.

To the best of our knowledge this is the first study to be conducted in Germany that examines whether CRC stage at diagnosis differs between migrants and non-migrants in an area of high migration (metropolitan area of Frankfurt am Main / Hanau / Offenbach, Germany). Furthermore, we will study possible explanatory factors for any differences that occur.

## Methods/Design

### Study design and setting

KoMigra is a cross-sectional, prospective observational study: during a 16-month consecutive recruitment process in the Frankfurt am Main / Hanau / Offenbach metropolitan area, data on tumour and migration status will be collected for 1,200 patients that have been newly diagnosed with CRC. Details on methods and design are laid down in the original study protocol, which can be requested from the corresponding author.

### Main and auxiliary questions

Is there a difference in the distribution of CRC tumour stage (based on the UICC (Union Internationale Contre le Cancer) classification) at diagnosis between migrants and non-migrants? What factors may explain any differences?

### Sample size calculation

The net sample size was fixed at 1,047 patients. The proportion of migrants in the study area is substantially higher than for Germany as a whole. The share is 43% for Frankfurt [17], 33% for Hanau and 55% for Offenbach [18]. However, migrants in Germany tend to belong to younger age groups: in Frankfurt, only 15.6% of the population aged 65 and over has a migration background [19]. According to the Schleswig-Holstein cancer register, the share of CRC patients in UICC stage I was 17% [20]. Assuming this share is only half as high among migrants (17% in the German population, compared with 8.7% for migrants), this difference can be detected with a significance level of ≤ 5% and a statistical power of 80%. To allow for possible losses, we plan the recruitment of 15% more patients and thus achieve a final sample size of 1,200.

### Sampling and recruitment, study timeline

In order to collect data from all persons that have been diagnosed with CRC independently of their origin and stage of disease, we will ask all providers of colonoscopies (gastroenterological private practices and certified centres for intestinal diseases [21]) in the region of study to participate.

Recruitment centres must conform to the following inclusion criteria: they must be located within a 20 km radius of one of the three city centres. The gastroenterological private practices must be officially permitted to carry out and charge for preventive and/or curative colonoscopies. The centres for intestinal diseases must be certified in accordance with German Cancer Foundation guidelines [21].

**Figure 1:**
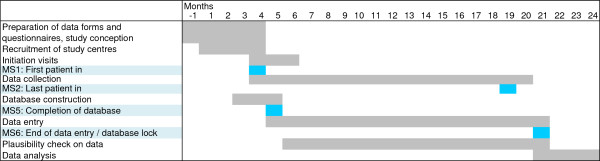
**Timeline of the KoMigra study.** MS = Milestone.

Nine certified centres for intestinal diseases and 68 qualified gastro-enterologists in private practice were identified and subsequently invited to participate in the study in an information letter sent out at the beginning of 2013. Starting from August 1, all recruitment centres will be asked for their participation agreement and instructed to initiate patient recruitment. During a 16-month period from August 1, 2013 to November 30, 2014, 1,200 patients with newly diagnosed CRC are expected to be consecutively included in the study. Recruitment centres will receive a compensation of 50 Euro for every CRC patient that is included in the study and whose documentation is complete. A timeline for the study is presented in Figure 1.

### Patient in- and exclusion criteria

To satisfy the inclusion criteria, a new and histologically confirmed CRC diagnosis during the previous three months is required, the patient must be resident in Germany and he or she must have provided valid consent to participate in the study. A patient will not be included if he or she has ever been previously diagnosed with another cancer (i.e. CRC is a second tumour; the only exception is basal-cell carcinoma).

### Data collection

At the recruitment centres, details of all CRC patients will be documented in a screening list. It assesses the in- and exclusion criteria of the patient and provides a complete record of all the presenting CRC patients during the recruitment period. If the patient fulfils all of the inclusion and none of the exclusion criteria, the study will be explained and he or she will subsequently be provided with an information sheet and informed consent will be obtained.

For all non-participants, the study personnel will note information on age, gender and tumour stage in the screening list as well as whether language problems were the reason for non-participation. In addition, the physician or health care assistant will provide information on the assumed migration background and country of origin.

The clinical tumour data form assesses details on CRC diagnosis, histomorphology of tumour and stage of disease, as well as patient risk factors (see Additional file 1). The CRC patient will be given a questionnaire with simple questions relating to socio-demographics and migration (see Additional file 2). The questions on the patient’s migration background are based on published methodological standards used in epidemiology research [22,23]. The migration background will be operationalised via the patient’s and his or her parents’ place of birth: Patients born outside Germany will be considered to be first generation migrants, while patients whose mother and/or father was/were born outside Germany will be considered to be second generation migrants.

In addition to the German version, the patient questionnaire, patient information sheet and informed consent will also be available in nine other languages that are common in the study region. The translation of the documents will be entrusted to a certified translation agency that can provide references in the field of clinical research.

All data will be monitored by study personnel during regular monitoring visits. Collected data will be promptly entered into a database at the Institute of General Practice to ensure they are recorded and kept securely. Data will be analysed in pseudonymous form. Before entries are made in the database, each patient will be checked to ensure he or she has not been documented by another centre, thus avoiding the duplication that can, for example, result when a gastroenterologist in private practice refers a patient to a centre for intestinal diseases. Personal data will be stored separately from identification features. Data collection will be concluded on December 31, 2014.

### Analysis

The statistical evaluation will consist of a descriptive and an analytical section. Categorical characteristics (such as country of birth) will be aggregated appropriately, so that if possible no category includes fewer than 10% of all test persons. In the descriptive section, the distribution of all factors collected from the questionnaires will be presented in two groups according to whether the test person has a migration background or not. The distribution of characteristics collected from non-participants will be described in the same way. Differences in the distribution of migrants versus non-migrants and participants versus non-participants will be compared using appropriate *χ*2-, t-, F- or Wilcoxon tests.

In the analytical section, the same variables will be subjected to explorative analysis by means of multivariable, logistic regression models, with the dependent variable being the dichotomized stage of the tumour (UICC stage I versus more advanced than I). Odds ratios and associated 95%-confidence intervals will be calculated. Furthermore, ordered logistic regression models will be estimated, with the exact stage of the tumour at diagnosis as the dependent variable. Predictors used in the ordered logistic regression will be both patient characteristics that are specific to migrants (how long in Germany, knowledge of German language, etc), as well as patient characteristics that are not specific to migrants (age, gender, socioeconomic status etc.). By including the first type of variable in the regression models, it will be possible to judge whether migration status remains influential in migrants that have been living in Germany for a long time, speak the language well etc. By including the latter type of variable in the regression models, it will be possible to judge whether migration status has an (additional) effect, after adjustment for variables whose distribution among migrants is different to that among non-migrants (socioeconomic status, age, etc.). Interaction models will be estimated in order to investigate whether the effects of patient characteristics on stage of tumour at the time of the initial diagnosis are different in migrants, compared to non-migrants. A detailed description of the statistical methods used in this study will be provided in a Statistical Analysis Plan (SAP) which will be finished before database lock.

### Process evaluation

The time frame and organisational rules of procedure laid down in the study protocol will be continually evaluated in order to ensure that they are adhered to.

All participating recruitment centres will be visited during the initiation phase and again during the first six months of the study period. Afterwards, visits will take place as required. The visits will be used to evaluate and ensure implementation of the study protocol. Documents that have been completed by then will be checked by study personnel for completeness and plausibility.

Should the screening lists reveal that only a small percentage of the total number of CRC patients are willing to participate in the study, then further study visits will be arranged in order to identify and eliminate any recruitment problems that may exist. If the participation rate and documentation from one recruitment centre differs from the levels achieved by the others, procedures will be discussed to establish reasons and potential solutions.

### Data quality assurance

The patient questionnaire, patient information and informed consent were piloted in all ten languages with regard to comprehensibility and applicability. The clinical tumour data form was piloted in the German language.

During the initiation visit, all persons involved in the study will be thoroughly trained and provided with the information required to conduct all steps in the study. Amongst other things, this includes identifying suitable patients, helping patients fill in the questionnaire on socio-demographic and migration data, and collecting data on those that are not willing to participate. During data collection, further training will take place where necessary. At least one physician and one health care assistant in every recruitment centre will be responsible for carrying out the study and will serve as contacts to answer any questions that may arise.

During the course of the study, plausibility checks will be carried out every two weeks by monitoring the number of migrants in the overall sample, the distribution of tumour stages, and the relative numbers of patients. Recruitment status will be checked by means of an interim analysis every three weeks in order to check that the targeted number of patients is reached via the ongoing recruitment process. In this way, measures to counter any problems that may develop can be implemented in good time (e.g., intensification of the monitoring process or the addition of further practices / centres for intestinal diseases).

### Avoidance of bias

Participation rates in studies are generally influenced by potential participants’ availability, ability to answer questions and readiness to cooperate. In the case of migrants, the factors ‘availability’ and ‘ability to answer questions’ (e.g. due to insufficient knowledge of German) present the greatest recruitment problems [24]. It is the aim of study recruitment to ensure that suitable measures are taken to counter well-known problems in order to keep selective participation to a minimum [25,26]: the translation of patient-relevant study documents into the nine most commonly spoken languages, as well as the use of simplified and easily comprehensible language in the patient questionnaire are two examples of these. Furthermore, the patient questionnaire should be filled in with the help of either the treating gastroenterologist or the health care assistant in order to enable illiterate and elderly patients, as well as patients with language difficulties to participate. The implementation of such measures aims to minimise any bias that may result from low participation rates among migrants.

In order to avoid systematic bias resulting from different care pathways and to achieve a representative sample with respect to the distribution of CRC tumour stages, CRC patients will be recruited from both gastroenterologists in private practice and certified centres for intestinal diseases throughout the Frankfurt / Hanau / Offenbach region.

Selection bias resulting from the aforementioned variables (e.g., higher participation of patients with low-stage as opposed to high-stage CRC) cannot be ruled out. It can, however, be assessed and described on the basis of the documentation of all participating and non-participating CRC patients in the screening list.

### Ethical approval and study registration

Ethical approval for the study was obtained from the leading Ethics Committee at the Frankfurt University Hospital on July 10, 2013. In Germany, ethical approval is needed for each federal state, in which participating study centres are located, not for each institution / participant. After approval is given, the information on the study conduction, the ethical approval and a list of participating sites needs to be transferred to the respective State Chamber of Physicians. This procedure has been fulfilled for the KoMigra study, and ethical approval from the State Chamber of Physicians was obtained at August 6, 2013. The study has been registered at the German Clinical Trials Register ([27]; DRKS00005056).

## Discussion

Our study investigates whether the distribution of tumour stage at the time of CRC diagnosis differs between migrants and non-migrants, while taking disparate migrant populations into consideration. If migration status proves to be an important predictor of CRC stage at time of diagnosis it should be included in common cancer documentation for purposes of health care research and quality improvement (e.g., by influencing early detection). A possible difference in tumour stage at diagnosis should also be investigated for other cancers.

It is planned that a second part of the project will use qualitative methods to take a more detailed view of the subject matter by means of qualitative interviews with migrants with CRC and their treating general practitioners (purposive sampling). It is necessary to find out about migrants’ pathways to diagnosis, as well as their feelings and experiences. Qualitative interviews will enable specific cultural features (e.g., body awareness, taboos), as well as social and other factors that are related to access to health care services, to be detected. This approach may also be helpful as relevant predictors such as ‘knowledge of the German language’ may be considered relevant on the basis of the quantitative survey, but in practice represent a culturally influenced combination of symptoms and other perceptions that, rather than language ability alone, is not expressed adequately in a German context.

Qualitative procedures may be better suited to taking a deeper look into such combinations of factors. The qualitative data will be used in addition to quantitative measures and thus be part of an explanatory and mixed-methods approach [28].

While the investigation of the study hypothesis in real-life is prone to several sources of bias, it has the potential to contribute valuable information on demonstrable health differences between migrants and non-migrants. The study question should also be extended to further cancer entities. The results could be directly included in modifications to corresponding cancer prevention strategies or as a basis for the development of new primary health care concepts for migrants.

## Abbreviations

CRC: Colorectal cancer; UICC: Union Internationale Contre le Cancer; SAP: Statistical analysis plan.

## Competing interests

The authors declare that they have neither financial nor non-financial competing interests.

## Authors’ contributions

AD, AS and CG designed the study. RvE and MB planned the statistical analysis. HZ was involved in the conception of the questions relating to socio-demographics and migration. AD and AS prepared the first draft of the manuscript. All authors have critically reviewed the manuscript for important intellectual content and given final approval of the version to be published.

## Pre-publication history

The pre-publication history for this paper can be accessed here:

http://www.biomedcentral.com/1471-2407/14/123/prepub

## Supplementary Material

Additional file 1 **Record of Clinical Tumour Data.** Questionnaire for assessment of details on CRC diagnosis, histomorphology of tumour and stage of disease, as well as patient risk factors. The form is filled by the responsible physician and / or health care assistant. Click here for file

Additional file 2 **Patient Questionnaire.** Patient questionnaire with simple questions relating to socio-demographics and migration. Click here for file
