# Supplementary material for: Colorectal cancer stage at diagnosis in migrants versus non-migrants (KoMigra): study protocol of a cross-sectional study in Germany
Source: BMC Cancer. 2014 Feb 24;14:123. doi: 10.1186/1471-2407-14-123 (PMC3939398; doi:10.1186/1471-2407-14-123)
Supplement: Additional file 1 — Record of Clinical Tumour Data. Questionnaire for assessment of details on CRC diagnosis, histomorphology of tumour and stage of disease, as well as patient risk factors. The form is filled by the responsible physician and / or health care assistant. [file 1471-2407-14-123-S1.pdf]

KoMigra

The Diagnostics of Colorectal Carcinoma in Migrants and Non-Migrants in Germany

Centre-ID			Patient-ID								

Record of Clinical Tumour Data

1. Diagnosis: ICD-10-Coding

C18<br>Malignant neoplasm of colon	C19<br>Malignant neoplasm of rectosigmoid junction	C20<br>Malignant neoplasm of rectum
☐	☐	☐

2a. Date of initial diagnosis *(= date of removal of tissue for investigative purposes)*

day		month		year			

2b. Reason for the diagnosis *(please check one box only)*

clarification of symptoms	early cancer recognition	long-term care CED /polyposis	follow-up	other / unknown
☐	☐	☐	☐	☐

2c. If for clarification of symptoms *(please check all relevant symptoms)*

occult blood	haemor-rhage	IBS irritable bowel syndrome	diarrhoea	obstipation	anaemia	weight loss	ileus
☐	☐	☐	☐	☐	☐	☐	☐

other: ....

3. Referred by

‚GP‘ (local doctor) ☐ specialist ☐ no medical referral ☐

4. Subsequent treatment by *(please indicate hospital or institution)*

....

not known ☐

5a. Histomorphological typing

adenocarcinoma	mucinous adenocarcinoma	signet-ring-cell carcinoma	carcinoid	other (please indicate histological type)
☐	☐	☐	☐	

5b. Histomorphological grading

G1 well differentiated ☐	G2 moderately differentiated ☐	G3 poorly differentiated ☐	G4 undifferentiated ☐

6a. Imaging procedures for disease stage ascertainment (tumour spread in the initial diagnosis)

(Please check appropriate box and indicate region of body examination date)

	☐ sonography	☐ CT	☐ MRT	☐ x-ray	☐ scintigraphy	other ________
**body region**						
**date**						

6b. TNM classification at initial diagnosis *(see list to be completed)*

c ☐ p ☐ T [ ]   c ☐ p ☐ N [ ]   c ☐ p ☐ M [ ]

6c. UICC stage at initial diagnosis

[ ]

7. Risk factors for CRC

**Risk factors**	**yes**	**no**	**unknown**
smoking	☐	☐	☐
alcohol consumption	☐	☐	☐
colorectal polyps	☐	☐	☐
inflammatory bowel disease (IBD)	☐	☐	☐
HNPCC / FAP	☐	☐	☐
positive family anamnesis (colorectal cancer in family)	☐	☐	☐

Thank you
