# Supplementary material for: Colorectal cancer stage at diagnosis in migrants versus non-migrants (KoMigra): study protocol of a cross-sectional study in Germany
Source: BMC Cancer. 2014 Feb 24;14:123. doi: 10.1186/1471-2407-14-123 (PMC3939398; doi:10.1186/1471-2407-14-123)
Supplement: Additional file 2 — Patient Questionnaire. Patient questionnaire with simple questions relating to socio-demographics and migration. [file 1471-2407-14-123-S2.pdf]

Centre-ID | | | Patient-ID | | | | | | | | |

Thank you for participating in our study!

Our study is aimed at improving the diagnosis and treatment of colorectal cancer.

Please complete this short questionnaire in your doctor's practice or in the centre for intestinal diseases (Darmzentrum) and return it to the practice nurse (MFA). Please use a ball-point pen to complete the questionnaire.

Please attempt to answer all questions truthfully. All information will be handled with the strictest confidentiality. It will not be disclosed to third parties and evaluated in such a way that the results make no reference to your person.

Even if you have no immigration history, please complete the questions on your origin as fully as possible. Although some of the questions may appear unimportant in your case, your answers are important for a sound result[1]

Your questionnaire begins on the next page! (please turn page)

To be completed by the practice nurse:

This questionnaire was completed *(please check as applicable)*

- ☐ Alone
- ☐ With help of the partner / person accompanying the patient
- ☐ With the help of the physician / nurse

Postcode and patient's place of residence: ……………………………………………………………………………………

Patient's height in cm: ………………………………………………….

Patient's weight in kg: ………………………………………………….

Questions concerning your person

Date

1. You are: ☐ male ☐ female

2. In Germany, who do you contact when you have a health problem?
(you may check several boxes)

☐ My 'GP' – general medical practitioner (local family doctor)
☐ A specialist medical practitioner
☐ I go directly to a hospital
☐ I treat myself
☐ Other: ………………………………………………………………………………

3. How much time elapsed between the first symptoms of colorectal cancer and the colonoscopy?

☐ I had no symptoms
☐ < 1 month
☐ 1 to 3 months
☐ > 3 to 6 months
☐ > 6 months

4. Have you ever had health problems whilst in Germany and consulted a medical practitioner (doctor) in your country of origin?

☐ yes ☐ no ☐ I do not come from another country

5. You live:

☐ With a partner or family
☐ Alone
☐ In a home for old people / nursing home / sheltered housing scheme
☐ ……………………………………………………………………………………………………….

6. How many persons are there in your household?

(please count yourself and all children)

Total ☐☐ persons

➔ How many are younger than 14? ☐☐ persons

7. How many years did you attend school (not counting vocational training college, university, etc.)?

☐☐ years

8. What is your highest educational (school-leaving) qualification?

(Please check the appropriate box)

☐ No final school final school leavers certificate / completed basic education only

☐ Statutory education (e.g. primary school, secondary school)

☐ General certificate of education at ordinary level, or secondary modern school leavers certificate (higher than statutory education, e.g. comprehensive, middle school, secondary school)

☐ Higher school leavers certificate / matriculation, general certificate of education at advanced level (lyceum)

☐ Other educational certificate: ………………………………………………………………………………

9. What is your highest professional qualification (including vocational training college, university, etc.)?

(please check the appropriate box)

☐ No occupational training certificate

☐ Apprenticeship, occupational training

☐ Polytechnic, engineering college

☐ Higher education school, university

☐ Other occupational training certificate: ………………………………………………………………….

10. Which of the following details apply to your occupational activity?

I am currently …

☐ … not gainfully employed (pensioner / student / housewife, etc.)

☐ … unemployed

☐ … employed part time or on an hourly basis

☐ … fully employed

11. What was or is your occupation?

Please write an exact occupational description on the line below (e.g. electrician).

………

12. In what occupational position are you currently-, or were you last active?

☐ Worker

☐ Self employed

☐ Employee

☐ Civil servant / justice / professional soldier

☐ Assisting family member

☐ Other: ……

13. How much total net income is available to your household per month?

Please include all income and social benefits (e.g. housing allowance, child benefits, unemployment benefit).
Net means the total amount after the deduction of tax and social security contributions.
Please check the appropriate box to indicate the amount of monthly net income.

☐ < 750 EUR

☐ 750 – 1,500 EUR

☐ > 1,500 – 2,500 EUR

☐ > 2,500 – 3,500 EUR

☐ > 3,500 EUR

Questions concerning your origin

14. In which country were you born?

………

15. Since when have you been living in Germany (including the former GDR)?

☐ Since birth ☐ Since | | | | | (indicate year)

16. In which country were your parents born?

Mother: ………...

Father: ……..

17. Do you have…?

☐ …German citizenship

☐ …unlimited right of residence

☐ …time-limited right of residence

18. What is your mother tongue?
(You may check more than one box)

☐ German

☐ English

☐ French

☐ Greek

☐ Italian

☐ Polish

☐ Russian

☐ Serbian

☐ Croatian

☐ Turkish

☐ Other, mother tongue is: ……………………………………………………………………..

19. How well do you speak German?
(please check the appropriate box)

☐ not at all ☐ not well ☐ fairly well ☐ well ☐ mother tongue

20. What is your religion?
(please check the appropriate box)

☐ Christian (catholic / protestant)
☐ Christian Orthodox
☐ Christian Free Church
☐ Jewish
☐ Muslim
☐ Buddhist
☐ Hindu
☐ No religion
☐ Other religion, please indicate: …………………………………………………………………….

21. What role does religious belief play in your daily life?

☐ no role ☐ modest role ☐ large role ☐ very important role

Thank you for your cooperation and help!
